# Return to play in elite rugby players after severe knee injuries

**DOI:** 10.4102/sajp.v78i1.1629

**Published:** 2022-04-21

**Authors:** Aneurin D. Robyn, Quinette A. Louw, Jochen Baumeister

**Affiliations:** 1 Department of Health and Rehabilitation Sciences, Faculty of Medicine and Health Sciences, Stellenbosch University, Cape Town, South Africa; 2 Department of Exercise and Health, Faculty of Science, Paderborn University, Paderborn, Germany

**Keywords:** performance, physical profile, preinjury level, return to play, knee injury, rugby

## Abstract

**Background:**

Medical professionals working in an elite sport environment have the challenging task to balance the athlete’s readiness to return to the playing field after severe injury with other stakeholders’ (coaches, sponsors, teammates) opinions and objectives.

**Objectives:**

Our study aimed to evaluate differences in the physical profiles of elite rugby players at return to play (RTP) after a severe knee injury, compared with their pre-injury profiles and matched controls.

**Method:**

Before the injury, participants performed four performance tests during their preseason screening. These tests were repeated and compared to baseline once a player was declared fit to play.

**Results:**

Significant differences (*p* ≤ 0.05) were found in the injured players’ group who were slower over 10 m speed, in their decision-making time and the total time of the reactive agility tests at RTP, whilst controls were significantly faster over 10 m and 30 m speed tests. The countermovement jump outcomes showed significant improvement in the uninjured participants (*p* ≤ 0.05).

**Conclusion:**

Our study highlights that injured players’ running speeds and decision-making times are slower after injury. The uninjured players have a positive outcome to training and match stimulus by improving their running speed and lower body explosive power during the season.

**Clinical implications:**

Our study provides insight into the RTP profile of elite rugby players, and a novel finding was the decision-making time deficit. This highlights the importance of cognitive training during injury rehabilitation as athletes make numerous decisions in a pressured and uncontrolled environment during a match. Speed training development is recommended as the athletes were slower after severe knee injury.

## Introduction

Rugby, a worldwide sport and one of the popular team sports in South Africa (Calvisi, Goderecci & Necozione 2016; Kaplan et al. 2008), is associated with a high injury occurrence (Yeomans et al. 2018). Rugby union records amongst the highest incidences of match injuries in all professional sports (Brooks & Kemp 2008). Rugby injuries are increasing and usually occur during a rugby match compared with training (Viviers 2018). In rugby union, injuries of the lower extremities are most prevalent compared with the rest of the body (Bird et al. 1998).

The knee joint is a frequent injury location, with the medial collateral ligament (MCL) injury being the most common and damage to the anterior cruciate ligament (ACL) being associated with more frequent days off until returning to play (Kaux et al. 2015). Knee injuries place the largest load on the medical team (Dallalana et al. 2007) as they lead to the greatest number of days in time spent on injury rehabilitation and medical attention. Severe knee injuries often result in extended periods of absence from play, affecting team selection performance and the overall health of an injured athlete who is being pressurised to return to play (RTP) (Hägglund et al. 2013).

When players are injured, they are expected to recover and perform at the same capacity as they were prior to their injury (Feucht et al. 2016). The athletes’ preparedness to RTP is influenced by several factors. Whilst there is prolific research about knee injuries (and specifically ACL injury as a model for a severe knee injury), studies regarding RTP after knee injuries in rugby union remain scarce (Sclafani & Davis 2016). There is no consensus regarding the RTP criteria post-injury (Menta & D’Angelo 2016). How long it takes to RTP differs according to the type and extent of the injury, demonstrating the complexity in reliably predicting prognostic outcomes and RTP timeframes.

An RTP assessment is a challenge for clinicians as the same type of injury can have different outcomes and RTP periods because of the interplay of several contextual factors. Recently, Bakshi et al. (2018) evaluated professional National Football League (NFL) players and concluded that the type of knee ligament injuries was the determining factor on RTP rates (Bakshi et al. 2018). Athletes with isolated ACL or a combination of ACL and MCL injuries have significantly quicker RTP than athletes who sustain other knee ligament injuries. With the advances in surgery and rehabilitation, professional athletes have a better chance of returning to their pre-injury level than previously (Myklebust & Bahr 2005). According to a recent consensus statement, RTP after ACL injury is defined by an athlete who returns to compete at a level comparable to before the injury (Meredith et al. 2020).

Return to play test batteries that have been described and typically used in sports teams include isokinetic testing, hop-, speed-, change of direction-, explosive lower body-, anthropometry-and psychological tests (Van Melick et al. 2016). The injured rugby players who return to the game will require lower body strength, speed, coordination and quick decision making. Therefore, a single test will not be sufficient for RTP criteria to predict a successful return or to minimise the risk of re-injury (Mueller, Bloomer & Durall 2013). However, only a few studies on ACL injuries have used objective criteria for athletes to RTP (Harris et al. 2014).

Competitive athletes who underwent ACL reconstruction and completed a test battery (isokinetic testing, four single-leg hop tests and two self-report outcome measures) significantly decrease the re-injury rate (Grindem et al. 2016). Ardern et al. (2016) mentioned in their consensus statement on RTS that ‘open skill situations’, which require athletes to react and make decisions when completing motor tasks should be incorporated in an RTS test battery (Ardern et al. 2016). Consequently, in our study’s sports-related environment, a reactive agility test to measure the sensory, motor and cognitive needs of a player upon returning from injury was included.

The limited knowledge base of research into RTP amongst rugby players warrants further investigation in this popular, dynamic contact sport. To our knowledge, there are no studies in elite rugby players that compare physical profiles at RTP with baseline after sustaining a severe knee injury. To address the knowledge gaps in RTP after severe knee injuries, our study aimed to (1) evaluate whether there are any differences in the physical profiles ([a] single-leg hop for distance, [b] countermovement jump [CMJ], [c] reactive agility time [RAT] and [d] speed [10 m and 30 m] tests) of elite rugby players at RTP compared with their pre-injury profiles and (2) compared with matched controls after a severe knee injury.

## Methods

A prospective cohort study was conducted in an indoor biomechanical laboratory and an indoor sports facility with an artificial grass surface. This kept the testing environment consistent by eliminating any weather condition’s influence.

Fourteen (*n* = 14) male, elite rugby players (18 years and older) from the Cape Winelands and Cape Metropole who had sustained a severe knee injury during the season either in a match or during training participated in our study. A severe injury was defined (per World Rugby Injury definition) as a player being unable to train for 28 days after sustaining an injury (Fuller et al. 2007). The knee injury diagnoses included (1) nine × ACL, (2) two × posterior cruciate ligament (PCL), (3) two × MCL and (4) one meniscal injury. The time loss of the 14 participants ranged between 2 and 10 months (mean 5.98 ± 2.92 months) from the date of injury until RTP.

Matching controls to injured participants were selected according to age, body mass, height and specific playing position. Table 1 shows the demographics of both injured and uninjured participants. The controls were part of the baseline testing group and therefore completed the same four physical performance tests as part of their preseason screening at the same time as the participants. These data were defined as their baseline assessments.

**TABLE 1:** Demographics of injured and uninjured participants (*n* = 28).

Variable	Injured	Uninjured	*p*
	Mean ± SD	Mean ± SD	
Age	20.5 ± 1.5	20.2 ± 0.7	0.151
Body mass	94.24 ± 14.02	94.10 ± 10.78	0.475
Height	181.814 ± 8.510	183.54 ± 7.67	0.142

**FIGURE 1:**
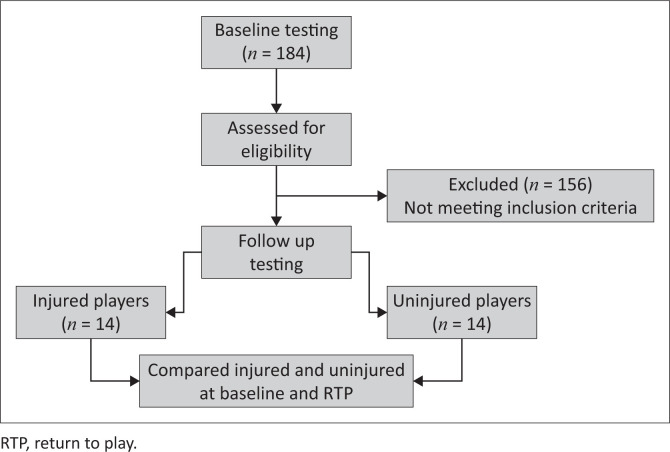
Consort diagram showing the key stages of testing.

We adopted a pragmatic approach to the sample size of injured participants. All elite, male rugby players (*n* = 185) in the Cape Winelands and Cape Metropole were tested at baseline. Due to the limited time and resources for our study (the study formed part of a three-year doctoral thesis), all players who experienced a severe injury (as described in the ‘participant eligibility’ section) during one season were included. Figure 1 shows the key stages of testing. Based on prior data obtained from the rugby union, we estimated that about 10–15 players would sustain severe knee injuries.

### Recruitment of all elite players for baseline assessment

The first author telephonically contacted the Head of Medical Services of the elite rugby teams in the Cape Winelands and Cape Metropole area and explained the purpose of our study. A follow-up email was sent with all the relevant study information. The interested and confirmed teams were contacted to discuss possible dates for baseline testing.

### Follow-up recruitment of injured players

The team doctor of each team informed the first author when a player was injured and met the eligibility criteria. The participant details were completed on the injury database and an identification number was allocated to each participant. The details included their age, body mass, height, team represented, playing position, injury diagnosis, estimated return to the training date and estimated RTP date. The first author telephonically contacted the participant to further explain our study and follow-up procedures.

The treatment and rehabilitation of the participant were the responsibility of the participants’ medical team and not influenced by the first author regarding return to train or RTP dates. When a participant was declared fit to RTP by their medical team, the first author was contacted to indicate the date of RTP. The matching controls were also identified depending on the injured participant profile. The controls’ follow-up testing was done on the same day as the injured participants’ RTP date and was compared with their baseline measurements.

### Outcome assessment

We included four outcomes in our study, these are provided in the following subsections.

#### Single leg hop for distance

The single leg hop for distance (SHD) test was initially conducted on the participants’ dominant limb, defined as the preferred kicking leg. This would provide the most accurate assessment of the participants’ strength and power in the limb that is most frequently utilised to adjust the body’s momentum whilst in contact with the ground (Frank et al. 2019). The participants stood with the tip of the big toe of one foot touching a piece of tape on the ground and performed a single hop on one leg in a straight-line landing on the same foot. The distance was measured in centimetres from the starting tape to the back of the heel upon landing. For each attempt, participants had to land without losing their balance. A failed attempt was logged if participants shifted the plant foot upon landing or touched down the second foot at any point during the hop or landing. One practice attempt on each leg was allowed before the SHD.

Each player performed three hops per limb with a 30 s rest break between attempts. The best jump distance of the three attempts was used to calculate the limb symmetry index (LSI) for analysis. The specific measures for the outcomes included in the analysis for the SHD were the hop distance in centimetres and hop distance symmetry in percentage between legs. The test–retest reliability of this standardised protocol has been established and well researched (Dingenen et al. 2019). LSI is calculated by the ratio of the two limbs (right and left). In healthy and uninjured participants, the calculation is the best distance jumped by the non-dominant leg divided by the best distance jumped by the dominant leg × 100. When an athlete returns to play the best distance jumped by the injured limb is divided by the distance jumped by the unaffected limb × 100 to calculate a percentage score (Abrams et al. 2014) and compared with baseline assessment.

#### Countermovement Jump

The CMJ was performed on a dual force plate (1016 × 762 × 125 mm, AMTI AccuPower, United States of America) sampling at 400 Hz. The force platform was linked to a portable computer, which was used to capture data. Force–time curves were created from the data obtained and calculated using a custom programme (AccuPower Software). The data were calculated relative to body weight as participants who were compared in the injured and uninjured groups had different body mass and playing positions. Asymmetry variables were chosen to evaluate any differences in inter-limb performances. Athletes’ CMJ’s performance to measure lower body explosive power is well researched (Eagles et al. 2015). Previous studies have highlighted inter-limb asymmetry when athletes return from lower limb injury from soccer (Hart et al. 2019), basketball (Heishman et al. 2019) and youth elite team-sports athletes (Fort-Vanmeerhaeghe et al. 2020). A CMJ performance on force platforms is regarded as the benchmark for test accuracy to measure lower-body power (Requena et al. 2012).

Before the testing sessions started, a thorough description of the testing procedures was given. After the standardised 15 min warmup (total body dynamic stretches, mobility drills and self-myofascial release), participants completed two submaximal practice jumps to adequately familiarise themselves before the jump testing. The test itself entails each participant completing three maximal CMJ with 3 min breaks in-between. All athletes were instructed to step on the force plate, place their hands on their hips and jump as high as they could when they were ready. The CMJ was performed without the use of the arm swing. In terms of reliability, the testing environment at baseline and RTP testing were consistent. Specific attention was necessary to ensure that no arm swing was used, that athletes maintained the hip, knee and ankle extension during flight and that players landed in the same position as they took off from (jump displacement).

The average of the countermovement jump performance variables data was used for analysis: The outcome parameters that were used were (1) jump height, (2) force at zero velocity, (3) peak force, (4) peak force asymmetry, (5) peak power, (6) peak power asymmetry, (7) rate of force development (RFD) and (8) reactive strength jump height, peak force asymmetry and peak power asymmetry. These outcome measures for the analysis of the CMJ have been highlighted as CMJ performance markers in other studies (McLean et al. 2010; McMahon et al. 2017).

#### Reactive agility time

Two-timing gates were positioned in a straight line at 0 m and 5 m, and another two were placed on either side of the course in a Y-formation (see Online Appendix 1, Figure 1-A1). Participants ran in a Y-pattern by running 5 m forward and then cutting to the left or the right at 45° when presented with a light stimulus. Participants started with their leading foot 0.3 m behind the first timing gate. When the participants crossed the 5 m timing gate, coloured lights (red, green, blue) were flashed either on the left or right timing gates signalling the direction in which the participant should run. Participants performed three trials of the RAT. A 3 min rest break was incorporated by allowing participants to take turns. The best trial (fastest total time) of the three repetitions was used for analysis. The specific measures for the outcomes included in the analysis for the RAT were (1) motor time in seconds (Split 1), (2) decision-making time in seconds (Split 2) and (3) total time in seconds (TT). The validity and reliability of this testing protocol have been established (Oliver & Meyers 2009).

#### Speed (10 m and 30 m)

Participants’ running speeds were tested over 10 m and 30 m. Electronic timing gates were used to measure participants’ speed performances and have been shown to be a reliable assessment device (Brown, Vescovi & Van Heest 2004). Athletes’ position can impact performance (Cronin et al. 2007), therefore the same starting position was always adopted, namely a split stance (one leg forward and 30 cm behind the starting line and one leg back). Timing gates were set at both the 10 m and 30 m marks to measure both acceleration and maximal velocity. The timer started once the player ‘broke’ the start timing gate and stopped after sprinting the full 30 m distance. Athletes were counted down (‘3 – 2 – 1 – GO’) and each player completed three sprints, each separated by a 3 min rest to ensure reliable results.

Each participant had three chances with the fastest time being recorded to the nearest 0.10 s. The best trial (fastest 10 m and 30 m time) of the three repetitions was used for analysis. The specific measures for the outcomes included in the analysis for the speed were 10 m and 30 m sprint time in seconds. Speed testing of rugby union players is reliable and is usually part of a standard test battery used for the physical profiling of players (Darrall-Jones et al. 2015).

### Procedures

**FIGURE 2:**
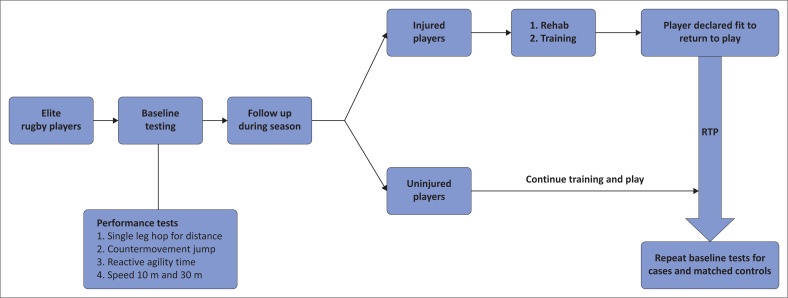
Return to play (RTP) testing procedure.

The baseline assessments (four physical performance tests) were completed during participants’ preseason screening (see Figure 2). This was done at the start of the rugby calendar before structured training and interventions started. All participants completed a 12-month injury history form and were declared medically fit by their team doctor before testing started. When the participants were declared fit to RTP at a level comparable to before injury by their medical team, the four physical performance tests were repeated within 1–2 days pending participant and testing venue availability. Participants completed a structured 15 min warm-up and then started with the RTP test battery.

Firstly, the SHD was measured on a non-slip, even surface, and thereafter the CMJ was performed on a dual-force plate at an indoor laboratory. Participants then went to a close-by indoor facility with an artificial grass surface to perform the RAT and the speed tests, respectively. Participants were wearing non-slip athletic shoes for the SHD and CMJ and their training boots for the RAT and speed tests. The same testers performed the physical assessments at baseline and RTP of all participants at the same locations. The testing venues and conditions were therefore consistent and not influenced by weather conditions.

The follow-up testing of the matched controls was on the same day as the injured player’s return to the play testing date. A comparison was possible between an injured and uninjured player as both players were tested on the same day with the exact timeframe between baseline and follow-up measurements. Feedback (within 24–36 h) comparing the RTP and baseline data was provided to the participant, and the medical team after testing was completed for each specific player.

### Statistical analysis

The collected data were entered into STATA 15.1 (StataCorp 4905 Lakeway Drive College Station, TX 77845, USA) and cleaned for analysis. At a 5% level of significance, the data for all analyses followed a normal distribution. We computed the Shapiro–Wilk test to assess the normality assumption of the data. Repeated measures ANOVA was completed for between-group difference (Table 3) of injured and uninjured at baseline and RTP. Paired *t*-tests and two-sample *t*-tests were performed to see whether there was a significant mean difference between (injured and uninjured at RTP and baseline) and within (injured at RTP and baseline and uninjured at RTP and baseline) players for different tests (RAT, 10 m and 30 m speed, single-leg hop for distance and CMJ).

### Ethical considerations

Our study was approved by the Stellenbosch University Health Research Committee (reference number: S18/01/010) and was conducted in accordance with the Declaration of Helsinki. All participants provided written informed consent in their preferred language before data collection.

## Results

### Baseline compared with return to play within groups comparison (*t*-test output)

Table 2 presents the within-group comparison of baseline measurements compared to RTP of the injured and uninjured participants. The injured participants’ performance was slower in three outcomes at RTP compared to baseline. Injured players were significantly slower in the 10 m speed test, decision-making (RAT Split 2) and the total time of the reactive agility (RAT total time) testing.

**TABLE 2:** Injured and uninjured performances at return to play compared with baseline (*n* = 28).

Performance Test	Injured		Uninjured	
	Mean difference (RTP – Baseline)	*p*	Mean difference (RTP – Baseline)	*p*
RAT split 1 (s)	0.009	0.728	−0.3	0.172
RAT split 2 (s)	0.088	0.043	0.053	0.214
RAT total time (s)	0.096	0.049	0.023	0.656
Speed 10 m (s)	0.048	0.034	−0.027	0.090
Speed 30 m (s)	0.048	0.116	−0.035	0.036
SHD (m)	−0.006	0.797	0.065	0.033

RAT, reactive agility time; RTP, return to play; SHD, single leg hop for distance.

The uninjured players at follow-up (same time as RTP of injured players) were significantly faster in the 10 m and 30 m speed test compared with their baseline times. The SHD of the uninjured participants also improved significantly at RTP compared with baseline.

Table 3 summarises the within-group comparison of CMJ baseline compared with RTP of the injured and uninjured participants. No significant changes were observed in the outcomes of the injured participants. The uninjured players showed marked improvement with CMJ performances at RTP in force @ zero velocity (kg), peak force (kg) and reactive strength index (net impulse).

**TABLE 3:** Countermovement jump performances of injured and uninjured participants at return to play versus baseline (*n* = 28).

Performance test	Injured		Uninjured	
	Mean difference (RTP-baseline)	*p*	Mean difference (RTP)-baseline	*p*
Force @ zero velocity (kg)	1.028	0.231	1.992	0.003
Jump height (cm) net impulse	−1.257	0.101	−1.271	0.227
Peak force asymmetry	−1.143	0.646	−0.642	0.672
Peak force (kg)	1.035	0.107	1.771	0.004
Peak power asymmetry	−3.285	0.182	−2.857	0.051
Peak power (kg)	−0.857	0.412	−0.771	0.558
Rate of force development (max)	1.771	0.248	3.728	0.101
Reactive strength index (net impulse)	1.285	0.569	5.121	0.004

RTP, return to play.

### Between-group differences at return to play and baseline (*t*-test output)

No statistically significant differences were noted between the groups at baseline, except for SHD as the injured players jumped significantly further at baseline compared with the control players (mean difference = 0.084 m; *p* = 0.001). Although there were no significant differences at RTP, the injured players were slower in five of the outcomes (see Online Appendix 1, Table 1-A1). No statistically significant changes were noted for any of the outcomes between-group differences of the CMJ performances at RTP and baseline (see Online Appendix 1, Table 2-A1).

### Between-group comparison of injured and uninjured with baseline and return to play (ANOVA)

**FIGURE 3:**
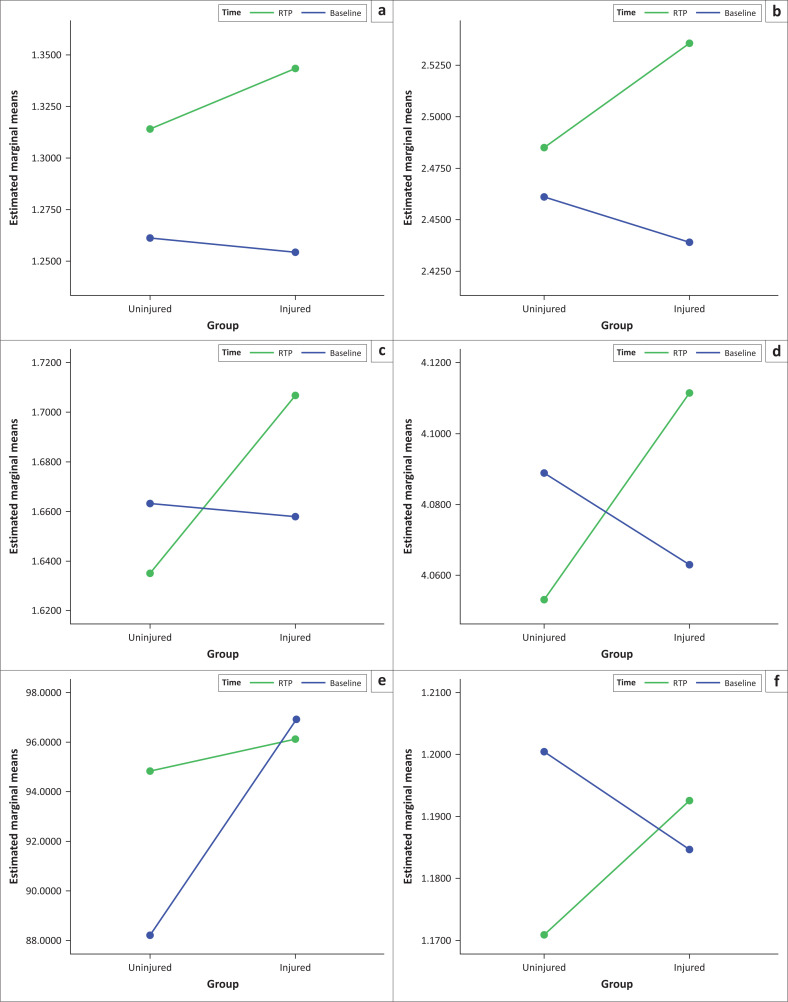
Graphs to illustrate group differences in physical performance at baseline and at return to play. (a) Estimated marginal means of reactive agility time (RAT)_split 2. (b) Estimated marginal means of RAT_total time. (c) Estimated marginal means of speed 10 m. (d) Estimated marginal means of speed 30 m. (e) Estimated marginal means of single leg hop for distance. (f) Estimated marginal means of RAT_split 1.

The repeated measure ANOVA findings (see Online Appendix 1, Table 3-A1) for between groups showed no significant differences, except for SHD as the injured group performed much better than the uninjured at baseline (4.9 ± 1.569; 95% confidence interval: 1.674 to 8.125; *p* = 0.004). Graphs to illustrate group differences are displayed in Figure 3.

Table 4 provides an overview and highlights the results of injured and uninjured participants’ performances. The statistically significant results are mentioned below for the two groups when comparing within and between at the two different time points (RTP and baseline).

**TABLE 4:** Summary table of the results (of injured and uninjured at return to play and baseline).

Variable	Results
Within-group difference of injured (baseline vs. RTP)	Slower at 10 m speed at RTP Slower decision-making and the total time of RAT at RTP
Within-group difference of uninjured (baseline vs. RTP)	Faster at 10 m and 30 m speed at RTP Better SHD improved LSI score at RTP Improved CMJ performance outcomes at RTP
Between-group differences at baseline	No statistically significant differences, except for SHD
Between-group difference at RTP	No statistically significant differences found

CMJ, countermovement jump; RAT, reactive agility time; RTP, return to play; SHD, single leg hop for distance; LSI, limb symmetry index.

## Discussion

Our study addresses an important gap by providing selective objective data on the physical profile of elite rugby union players who sustained severe knee injuries at RTP and compared them with matched controls. Recent evidence suggests that athletes have decreased physical performance and a high injury rate upon RTP (Dickerson et al. 2020).

Our key findings were that the injured players were significantly slower in the 10 m speed, decision-making time and the total time of the reactive agility tests at RTP compared with their baseline assessments. Current studies report indifferent results regarding the athletes’ speed performance after lower limb injuries. Athletes in team sports like the National Basketball Association (NBA) and NFL had no differences between ACL injured players and matched controls on speed, agility and jumping parameters (Keller et al. 2015; Mehran et al. 2016). Although these parameters can vary according to player positions and demands as running backs, receivers, linebackers and defensive backs (speed-positions), a significant decrease at RTP following knee surgery is seen in NFL athletes (Aune et al. 2014).

Rehabilitation specialists are unlikely to delay an athlete’s RTP because of poor speed testing performance or insufficient training load; hence this might lead to increased injury risk and even decreased match- and team performances upon return (Stares et al. 2018). Speed training is usually introduced as part of the final phase rehabilitation programme after good movement patterns and strength are achieved (Schache et al. 2014). Therefore, speed training development is recommended after regaining strength, balance and motor control (Malone et al. 2018; Reiman & Lorenz 2011). Supervised physiotherapy sessions could have a positive impact on athletes’ speed and agility performances (Królikowska et al. 2018).

When their follow-up testing was compared with baseline times, the uninjured players were significantly faster over 10 m and 30 m speed tests than the players who suffered a severe injury during the season. The improvement of an athlete’s speed performance during the season has been reported (Thomas, Mather & Comfort 2014). This may be possible as uninjured players’ adaptation to training stimulus and periodisation during the season might increase speed performances. The uninjured athletes’ exposure to adequate strength training during the season improves jumping and running performances and makes this development in speed possible (Silva, Nassis & Rebelo 2015).

The injured group had statistically slower decision-making and total time at RTP compared with baseline. The decrease in this physical performance parameter could be because of the type of training during rehabilitation or the volume and type of team training before RTP. Recently, Marques et al. (2020) highlighted that RTP decisions do not usually include agility assessment as a criterion for the athlete to return to the playing field (Marques et al. 2020). Recent soccer studies emphasise the need for change of direction drills (COD) and agility training as part of injury rehabilitation (Dos’Santos et al. 2019) and that elite athletes improve their agility and speed with a dedicated programme (Naghavi et al. 2017).

Reactive agility drills are usually incorporated into the last phase of rehabilitation; therefore, sports medicine personnel must be encouraged to add and focus on this component. Another reason for the decreased reactive agility performance could be that (during the rehabilitation phase) athletes are exposed to COD drills and not reactive agility drills that are diverse and require visual stimulus and decision-making components. The incorporation of small-sided games during reactive agility performance has a positive effect on decision-making time (Young & Rogers 2014). When a player returns to play during the season, there are minimal small-sided games as part of the training, and therefore the rehabilitation team needs to incorporate these during rehabilitation and even to continue with extra reactive agility training although the player has returned to the playing field. An injured player returning from injury will only become better and reduce re-injury risk with more exposure to match-like training load and scenarios (Blanch & Gabbett 2016).

We found significant differences in the SHD amongst the uninjured that was unexpected. Their poor SHD performance at baseline was not expected as all participants were injury free and medically fit to play. We suspect that the reason for this significant improvement was that their baseline scores were below the LSI norm of 90% (Thomeé et al. 2011), and then they scored much better on the follow-up testing. At the follow-up testing, the LSI returned to above 90%, and therefore the statistically notable difference between follow-up and baseline measurements was observed. No other valid explanation could be found as the testing surface, tester and equipment were consistent throughout our study.

The CMJ indicated that although there were no statistically significant differences between the injured and uninjured participants comparing RTP and baseline measurements, the uninjured group improved statistically significantly in terms of force at zero velocity (kg), peak force (kg) and reactive strength index (net impulse) during the same period.

The CMJ was performed on a dual platform to evaluate limb symmetry at RTP, which is important for players who are returning from lower limb injury. There were no statistical differences regarding asymmetry in the injured group variables at RTP compared with baseline, which indicates a good rehabilitation outcome as asymmetry is usually present after lower limb injuries. Studies have shown that asymmetry is relevant and does exist when players RTP (Fort-Vanmeerhaeghe et al. 2020; Hart et al. 2019; Heishman et al. 2019). Recently (2018), multidirectional sports athletes who were at least 6 months post-ACL surgery displayed significant asymmetry values with single-leg CMJ outcomes amongst injured athletes and not amongst the control group (O’Malley et al. 2018). This emphasises that rehabilitation should address the asymmetry between injured and uninjured limbs as this could lead to compensatory movement patterns and future re-injuries (Maestroni et al. 2020). It is critical to remember that players that RTP with inter-limb imbalance can sustain a re-injury or a secondary muscle injury if the deficits are not addressed during rehabilitation. We showed no significant statistical differences in the injured group when comparing RTP and baseline measurements, thus complimenting the rehabilitation programme run by the various professionals.

The very positive sign of no inter-limb asymmetry is backed up with another as there was no significant difference of the other CMJ outcomes at RTP compared with baseline (Table 3). This is not the norm when assessing explosive lower body power after lower limb injury. Athletes that RTP after musculoskeletal injuries have decreased performance in maximal strength, rate of RFD and reactive strength (Maestroni et al. 2020). A study of elite soccer players confirms that although players returned to play, there was a decrease in force production, asymmetry and compensatory movement patterns amongst these players (Hart et al. 2019). In further confirmation, a recent study (2020) evaluated college athletes with ACL injuries and findings highlighted decreased force and increased asymmetry of the affected limb and that the lack of performance of these outcomes can contribute to ACL re-injury (Dai et al. 2021).

In summary of the CMJ performances, it is important to have pre-injury baseline scores to compare athletes with themselves so as to assist with RTP decision-making. The injured players did extremely well not to present with any asymmetry or decrease CMJ performance outcomes at RTP. In contrast, the uninjured players improved significantly during the season when comparing their follow-up testing with baseline testing. It is therefore concluded that the uninjured players are much improved from preseason because of specific training and match exposure.

## Study limitations

The sample size is limited because of the elite population. The reason for the limited participants is that according to the South African (national) rugby injury database there are two to three severe knee injuries per team in a season. Testing the other elite teams within South Africa would increase the sample size but access to the players, staff, testing equipment and facilities is a challenge. The inclusion of an aerobic test as part of the test battery would have been beneficial as this would enhance the physical performance profile of the participant at RTP. Although the injury – and RTP dates were available to the first author, the details of the rehabilitation programme, period (days) of rehabilitation, conditioning plan and training exposure are all factors that were not reported and may influence their performance. Finally, there was not a longer follow-up period of participants to see whether any of the outcomes are linked to their match performance.

## Conclusion

Our study highlights that injured players’ running speed and decision-making time are slower after a serious knee injury at RTP. The slower decision-making by a player after injury may result in decreased match performance and an increased risk of injury. The uninjured athletes have a positive outcome to training and match stimulus by improving their running speed and lower body power during the season. There have been recent RTP consensus statements, but there are still many questions about when an athlete can safely RTP and what is the ideal discharge criteria. Medical professionals working in an elite environment make daily decisions regarding RTP although it is a challenging task to balance the athlete’s preparedness to return to the playing field and all other stakeholders’ (coaches, sponsors, teammates, etc.) opinions and objectives. As Dingenen and Gokeler (2017) suggest shared decision-making amongst all the stakeholders and a criteria-based approach are preferred during the rehabilitation process compared with the traditional time-based process (Dingenen & Gokeler 2017).

## Clinical implications

Our results provide insight into the RTP (physical) profile of elite rugby players after sustaining a severe knee injury. A novel finding was the decision-making time deficit of injured players at RTP. This highlights the importance of cognitive training during injury rehabilitation as athletes make numerous decisions in a pressured and uncontrolled environment during a match. Speed training development is recommended after regaining strength, balance and motor control as the athletes were slower after a severe knee injury (Malone et al. 2018; Reiman & Lorenz 2011).

Although there were no significant differences between injured and uninjured participants at RTP (Online Appendix 1, Table 1-A1 and Table 2-A1), the uninjured participants improved remarkably during the season in various performance markers whilst the injured did not (Table 2 and Table 3). Another positive finding was that the injured players had no inter-limb asymmetry at RTP that contradicts findings of similar studies (Dai et al. 2021; O’Malley et al. 2018). A possible reason for this positive finding might be that elite athletes have dedicated sport medicine professionals to assist with their injury rehabilitation and adequate time to address their injury needs. Although injured players returning to preseason baseline are encouraging, this might not be good enough compared with positive progress by their uninjured counterparts.
